# Lay media reporting of rosiglitazone risk: extent, messaging and quality of reporting

**DOI:** 10.1186/1475-2840-8-40

**Published:** 2009-07-24

**Authors:** Doreen M Rabi, Adriane M Lewin, Garielle E Brown, Alun L Edwards, Jeffrey A Johnson, William A Ghali

**Affiliations:** 1 Department of Medicine, University of Calgary, Calgary, Canada; 2 Department of Community Health Sciences, University of Calgary, Calgary, Canada; 3 Department of Cardiac Sciences, University of Calgary, Calgary, Canada; 4 School of Public Health, University of Alberta, Edmonton, Canada

## Abstract

**Background:**

A meta-analysis suggested the use of rosiglitazone was associated with an increased risk for cardiovascular (CV) events. Rosiglitazone remained available for use as more definitive safety trials were ongoing. This issue was reported in the lay media.

**Objective:**

To review lay media articles to determine the extent of media coverage, the nature of the messaging, and to assess the quality of reporting.

**Methods:**

The Factiva media database was used to identify articles published between May 18 and August 31, 2007. Two reviewers (a lay person and a physician) screened full text articles for eligibility, appraised the articles for their tone (worrisome, neutral, not worrisome), and for the quality of medical data reporting.

**Results:**

The search identified 156 articles, 95 of which were eligible for our review. Agreement between the lay and medical reviewers in the appraisal of the article tone was 67.4%. Among those with agreement, the articles were often appraised as "worrisome" (75.3%). Among those with disagreement, the lay reviewer was significantly more likely to appraise articles as worrisome compared to the medical reviewer (77.4% vs. 3.2%, X2 = 9.11, P = 0.003). Cardiovascular risk was discussed in 91.6% of the articles, but risk was often reported in qualitative or relative terms.

**Conclusion:**

There were many lay media articles addressing the safety of rosiglitazone, and the general messaging of these articles was considered "worrisome" by reviewers. Quality of risk reporting in the articles reviewed was poor. The impact of such media coverage on public anxiety and confidence in treatment should be explored.

## Introduction

The lay media have become an important means for disseminating health information [1-4]. It has been demonstrated that media coverage related to medical therapy significantly impacts how such therapies are perceived and utilized [2]. However, it also been demonstrated that there are clear deficiencies in the reporting of health related stories in the media, and that less then half of medical therapy articles in the lay media report therapy related risks in a satisfactory manner, and only a third have satisfactory reporting of treatment benefits [5]. Given the impact the lay media has on medical decision-making, it is important to continually monitor the quality of the medical news reporting.

In May of 2007, Nissen and colleagues published a systematic review and meta-analysis examining the effect of rosiglitazone on cardiovascular outcomes, specifically it's effect on risk for myocardial infarction and all cause mortality [6]. This review pooled data from 42 studies and provided data on 27 847 patients. While the overall event rate for MI and death from any cause were low (158 events in 26011 persons, and 61 events in 20445 persons respectively), the authors did document a statistically significant increase in the risk for MI among those using rosiglitazone relative to those using a comparator agent (either another oral hypoglycemic or placebo). There was also an increase risk of death in the rosiglitazone group relative to the comparator group, however this was not found to be significant.

Diabetes and heart disease are highly prevalent conditions and rosiglitazone was a medication that was increasingly being used to treat diabetes and in theory, by reducing insulin resistance, to reduce the risk of cardiovascular disease. Therefore, there was wide general interest surrounding the results of this meta-analysis. We conducted a content analysis of lay media articles indexed in an international media database and specifically evaluated articles discussing rosiglitazone and sought to document the extent of coverage and the quality of reporting. The objective of this study was to review the content of lay media articles to determine how risk was communicated to the public.

## Methods

### Search Strategy

The Factiva media database was used to identify potentially eligible print media articles. Factiva is an international database that provides access to print, television and radio media from over 152 countries and uses a unique system of subject headings. A search strategy was developed in consultation with an academic librarian to ensure capture of all potentially relevant articles. Factiva indexing makes simple keyword searches most effective and we identified all articles containing the keywords "rosiglitazone" or "Avandia", published between May 18 and August 31, 2007. This search was restricted to English language articles.

### Inclusion and Exclusion Criteria

Two reviewers (DMR, GB) screened full text articles for eligibility. Articles were eligible for inclusion if they discussed heart or cardiovascular disease OR safety, AND rosiglitazone. Articles were excluded if they did not address the safety of rosiglitazone, or if the article's primary focus was on the impact of the Nissen article on rosiglitazone's manufacturer's (GlaxoSmithKline) stock prices.

### Measures of Interest

Two reviewers (one lay reviewer [GB], one medical reviewer [DMR]) appraised the articles for their tone. The tone of the article was graded based on the degree of context provided with the data reported on harm. Contextualization of risk has been deemed an important aspect of medical reporting [5]. The tone grade provided by the reviewers does not reflect the actual data reported in the original meta-analysis, but rather, how the data was presented in the media article. Tone was graded as worrisome (risk of harm reported, potential benefits of treatment and limitations of meta-analysis were not reported), neutral (risk of harm reported, either potential benefits of treatment and/or limitations of meta-analysis reported), or not worrisome (risk of harm reported but focus of article was benefits of treatment and limitations of meta-analysis). The reporting of risk (relative, absolute or not specified) was documented. Other indicators of quality of data reporting were also extracted. We employed a quality of reporting checklist that was adapted from Cassels et al [5]. These indicators included the use of generic (rosiglitazone) and trade (Avandia™) names; reference to the class of medication (thiazolideindiones or TZDs) or other medications in the class (pioglitazone); whether the original article was cited and whether sufficient information was provided to locate the original articles (ie could a lay reader conceivably find the original scientific article to review); was expert opinion reported (experts could not include the author) and if so, did experts disclose any potential conflicts of interest; and were any additional information resources provided. Data were extracted in duplicate and agreement between the reviewers was determined.

### Analysis

All extracted data were reported as proportions. Editorial articles were analyzed separately from non-editorial articles on the measure of article and title tone. Overall agreement between reviewers was caluculated using the Kappa statistic. When applicable, differences in proportions were tested using Chi square analysis.

## Results

**Figure 1:**
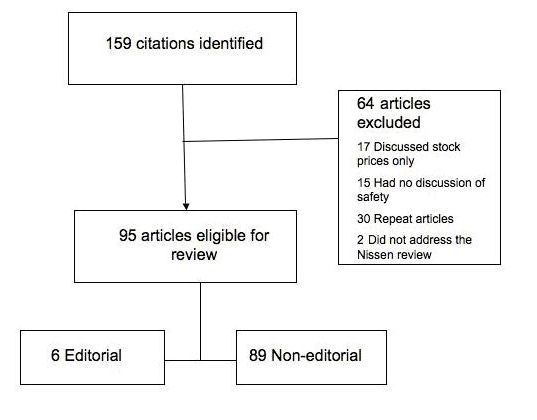
**Study flow chart for article selection**.

The search strategy yielded 159 original citations. Review of these articles led to the exclusion of 64 articles (see figure 1) and 95 articles were included in our content analysis.

### Tone of Articles

#### Editorial Articles

Six of the 95 eligible articles were editorials. There was 100% agreement between reviewers with respect to the evaluation of tone in these editorials. The titles were considered "worrisome" in 3 of the 6 editorials, and "neutral" in the remaining 3 articles. The content of the articles were considered more negative, with 4 of the 6 articles being graded as "worrisome", 1 being graded as "neutral" and 1 being graded as "not worrisome" (see Table 1).

**Table 1:** Categorization of overall tone (messaging) for the Editorial (n = 6) and non-Editorial articles (n = 89) identified

**Type of Article**	**Tone**		
	**Not Worrisome**	**Neutral**	**Worrisome**

*Editorial (n = 6)*			

Title	0	3 (50%)	3 (50%)

Article	1 (16.7%)	1 (16.7%)	4 (66.7%)

*Non-Editorial (n = 89)*			

Title	13 (14.6%)	24 (27%)	52 (58.4%)

Article	9 (10.1%)	13 (14.6%)	67 (75.3%)

#### Non-editorial Articles

Eighty-nine of the articles identified were non-editorial articles. Agreement between the lay and medical reviewer was quantified with a Kappa of only 0.579. Among the articles with agreement, the titles and articles were most often appraised as "worrisome" (58.4% and 75.3% respectively) (see Table 1). Among those with disagreement, the lay reviewer was significantly more likely to appraise the titles (58.1% vs. 35.5%, X^2 ^= 5.13, P = 0.02) and articles (77.4% vs. 3.2%, X^2 ^= 9.11, P = 0.003) as "worrisome" compared to the medical reviewer.

### Quality of Reporting

Data regarding the quality of reporting is detailed in Table 2. The concept of cardiovascular risk was explicitly addressed in 91.6% of the articles (editorial and non-editorial inclusive). Over half (58.6%) of the articles expressed risk in numeric terms, the remainder (41.4%) discussed it in qualitative terms. When risk was expressed numerically, 98% of the articles reported relative risk, but only 4.2% of the articles actually stated whether the risk being reported was relative or absolute in nature. However, the reporting of relative risk was not significantly associated with the appraisal of the article (X^2 ^= 0.55, P = 0.97).

**Table 2:** Measures of quality of reporting

**Quality Indicator**	**Present n/N (%)**
*Reporting of Risk*	

Cardiovascular risk clearly reported?	87/95 (91.6)

Reported risk quantified?	51/87 (58.6)

Nature of risk (absolute vs. relative) clearly stated?	2/51 (4.2)*

*Duality of Interest*	

Was an expert consulted?	13/95 (13.7)

If an expert was used, was there disclosure of potential conflict of interest?	0/13 (0)

*Citation of Sources*	

Was the review by Nissen et al. cited?	70/95 (73.7)

Could the Nissen review be found based on information provided in the news report?	61/70 (87.1%)

Were additional information resources provided to readers?	12/95 (12.6)

*Use of trade vs. generic drug names*	

Were both generic and trade names used in the report?	95/95 (100)

Was reference made to the class of medication (TZD)?	29/95 (30.5)

* All articles that did not specify the nature of risk reported, reported the cardiovascular risk in relative terms.

We found that all articles used both generic and trade names. Reference to the class of TZD medications was made in 69.5% of the articles and specific mention of the other TZD medication, pioglitazone, was made in 57.9% of the articles. Expert opinion was reported in only 13.7% of articles, but whether this expert had any potential conflicts of interest was never reported. The original article by Nissen et al. was explicitly mentioned in 73.7% of articles, and among those referencing the article, sufficient information to find the article for personal review was found in 87.1% of the articles. Additional sources of information were seldom provided to the reader (12.6%), and the most common resource provided to readers was the American Food and Drug Administration (FDA) website.

## Discussion

This review shows that there was indeed wide coverage of the meta-analysis by Nissen et al. [6] in the lay media. The general messaging expressed in these articles was considered "worrisome", particularly by the lay reviewer, and the overall quality of reporting was modest. These articles did use generic and trade names when referring to rosiglitazone, and they frequently cited the original scientific article. However, the reporting of risk was poor. Risk was discussed qualitatively in 41.4% of the articles, and when described in a quantitative manner, relative risks were used. In our study, an article was classified as worrisome if harm was discussed without placing the data of harm in some context. As many readers of these lay media articles will not read the original scientific article directly, the media, holds considerable responsibility to produce balanced reports on the quality of the original article, along with presenting risk in both absolute and relative terms. This review suggests that such appraisal was rarely done.

Perhaps the most interesting finding from our review is that when disagreement in the appraisal of article tone occurred, the lay reviewer consistently sensed more alarm in the title/article than the medical reviewer. Even when there is an attempt to provide data on both benefit and harm, the lay reviewer was more likely to attend to the message on harm. Given the prevalence of diabetes around the world, the degree to which this media coverage provided patients with valuable information about their treatment and the degree to which it caused heightened anxiety should be evaluated further.

While the results from the Nissen review raised concern, rosiglitazone remained available for clinical use as clinical practice leaders and drug regulators felt there were clear limitations to the Nissen review, and that there was insufficient data to say that the drug should be withdrawn. The meta-analysis included data from trials that were not designed to examine cardiovascular safety, as such, these trials did not have time-to-event data nor were all the cardiovascular outcomes adjudicated. Nissen also excluded trials with no cardiovascular events thereby introducing some bias into the review.

Market reports indicate that the sales of rosiglitazone dropped by $290 million in the United States in the months that followed the publication of this paper [7]. It would appear that irrespective of the quality of the scientific data, public and professional confidence in this therapy was compromised. A recent study by Shah et al. similarly demonstrated that there was significant decrease in new rosiglitazone prescriptions immediately following the release of the Nissen review [8]. Given emerging evidence on other adverse effects of TZDs such as osteoporosis and heart failure [9,10], whether the decreased use of rosiglitazone is entirely attributable to the media coverage surrounding the Nissen review is not known. Further, whether this change in practice was physician driven (due to a re-evaluation of the evidence regarding relative benefits and harms of rosiglitazone) or patient driven (possibly related to concerns raised in the media) is unclear.

The lay media will continue to be an important knowledge translation vehicle as it broadens communication of new health information. However, this study highlights that information on harm is perceived differently among the medical and lay readers, and we suspect that these differences in perceptions are in large part related the quality of reporting. Ongoing quality assessments of medical reporting are required to ensure that the messages communicated are truly informative to lay and medical audiences alike.

## Competing interests

The authors declare that they have no competing interests.

## Authors' contributions

DMR conceived, designed, conducted and performed all statistical analyses for the study, and was the lead writer on the manuscript. AML conceived the study and was lead editor on the manuscript. GB assisted in the conduct of the study. JAJ, AEL and WAG were senior editors and provided content expertise essential to design of the study. All authors approved the final manuscript.
